# Reinforcing Deep Learning-Enabled Surveillance with Smart Sensors

**DOI:** 10.3390/s25113345

**Published:** 2025-05-26

**Authors:** Taewoo Lee, Yumin Choi, Hyunbum Kim

**Affiliations:** Department of Embedded Systems Engineering, Incheon National University, Incheon 22012, Republic of Korea; hst0092@inu.ac.kr (T.L.); dbals28@inu.ac.kr (Y.C.)

**Keywords:** deep learning, surveillance, smart sensors, mobile

## Abstract

It is critical to solidify surveillance in 3D environments with heterogeneous sensors. This study introduces an innovative deep learning-assisted surveillance reinforcement system with smart sensors for resource-constrained cyber-physical devices and mobile elements. The proposed system incorporates deep learning technologies to address the challenges of dynamic public environments. By enhancing the adaptability and effectiveness of surveillance in environments with high human mobility, this paper aims to optimize surveillance node placement and ensure real-time system responsiveness. The integration of deep learning not only improves accuracy and efficiency but also introduces unprecedented flexibility in surveillance operations.

## 1. Introduction

In the contemporary landscape of global security, the deployment and advancement of surveillance systems have become paramount in safeguarding public and private spaces alike. The necessity of these systems extends beyond mere observation, playing a crucial role in preempting potential threats and ensuring the safety of individuals in various environments [1,2,3]. Also, it is critical to monitor wide areas and complex areas, which consist of numerous heterogeneous devices, with smart sensors, the Internet of Things (IoT), and the Industrial Internet of Things (IIoT) [4,5,6]. The barrier concept for surveillance was introduced by Kumar et al. [1].

**Figure 1:**
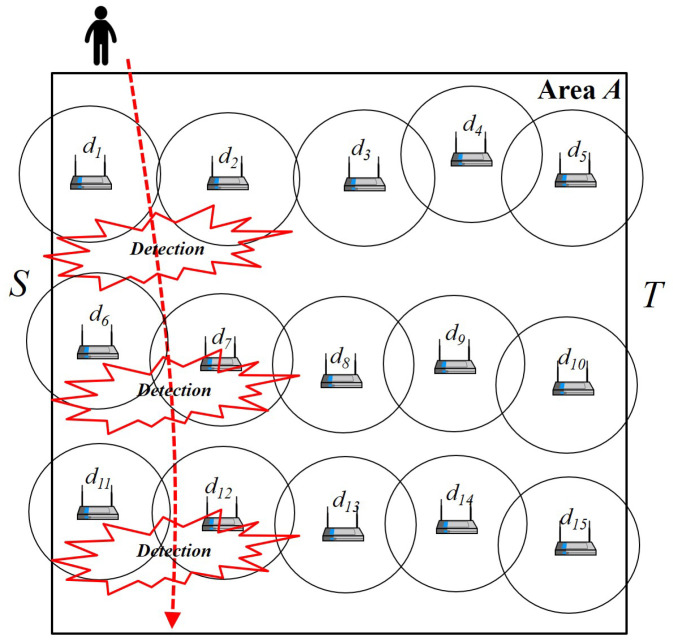
The barrier concept and penetration detection in the given area.

**Figure 2:**
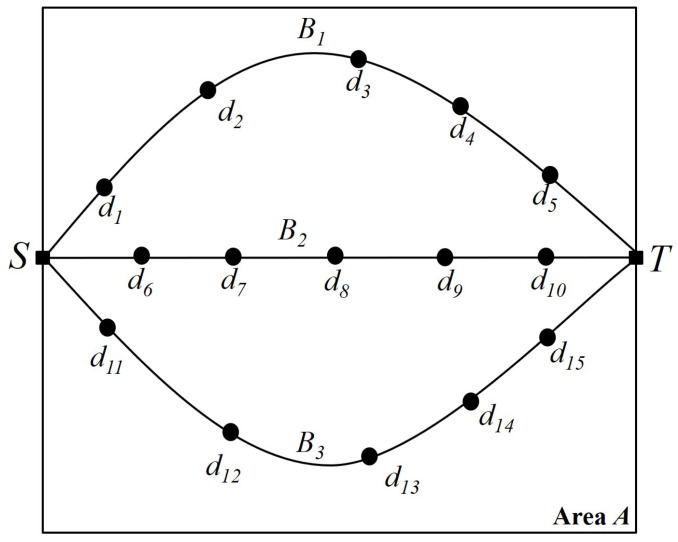
Graph representation of a barrier and its transition from a device.

**Figure 3:**
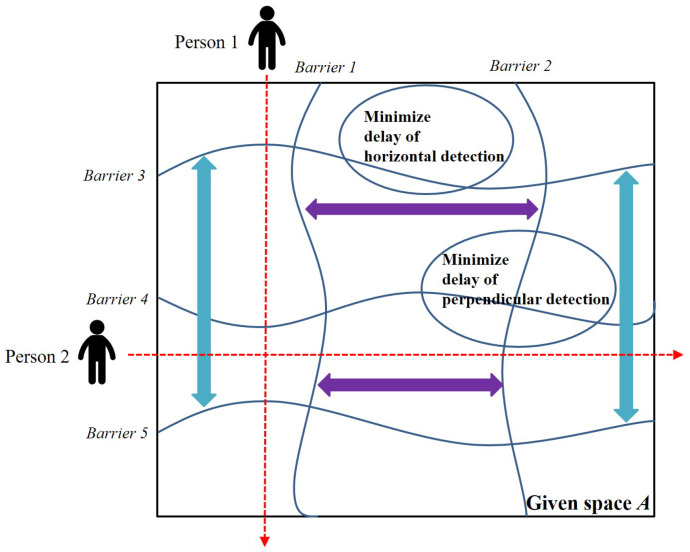
The two-way detection-enabled barrier example.

Figure 1 shows the barrier concept and penetration detection in the given square-shaped area *A*, called the *K-barrier*, where *K* is the required number of barriers to be formed in the area. As seen in Figure 1, there are three independent barriers, each composed of heterogeneous devices with wireless sensing ranges. With these barriers, it is guaranteed that any penetration from top to bottom or from bottom to top is detected by at least one sensor in the barrier. Figure 2 depicts the graph representation of a barrier and its transition from a device. Given area *A*, there is a set of devices $D=\{d_{1},d_{2},d_{3},...,d_{15}\}$. From source *S* to destination *T*, we can search for the possible maximum number of independent paths or node-disjoint paths between *S* and *T*. Each independent path can be considered one barrier, so we have three different barriers $B_{1},B_{2},$ and $B_{3}$. Also, Figure 3 depicts the two-way detection-enabled barrier example of minimizing the maximum delay of horizontal and perpendicular detection when mobile objects go into the given space. As can be seen, the maximum delay for mobile objects can vary according to the positions of the multiple barriers created.

The limitations of the conventional K-barrier model are evident in environments characterized by high levels of human mobility. These settings, where the patterns of movement are neither static nor predictable, necessitate a surveillance approach that can dynamically adjust to changing conditions. The static assumptions inherent in traditional K-barrier models are insufficient for such environments, as they fail to account for the variability and complexity of human traffic, which can significantly impact the effectiveness of surveillance operations. Traditional models, such as the K-barrier approach, provide a foundation for the development of surveillance systems but fall short in addressing the complexities of modern, dynamic environments. This is particularly true in public spaces such as airports, train stations, and shopping centers, where the dynamics of human movement present unique challenges to maintaining a robust surveillance framework [7,8,9,10].

Recent advancements in artificial intelligence (AI) and machine learning (ML) have demonstrated significant potential in enhancing the adaptability and efficiency of surveillance systems in resource-constrained circumstances. These technologies have been increasingly recognized as crucial in the development of smart surveillance systems, capable of autonomously adjusting to changes in their operational environment [11,12,13,14]. In particular, the recent advances in deep learning technology, with its unparalleled ability to process and learn from large datasets, offer a promising solution to the limitations of traditional surveillance models. So, the integration of deep learning into surveillance systems represents a significant shift toward more intelligent and responsive security measures. Also, it is highly necessary to devise a promising deep learning-based framework not only to improve the accuracy and efficiency of surveillance systems but also to cover a level of flexibility previously unattainable with traditional models. And, we should consider various factors that influence the dynamics of public spaces, including but not limited to human movement patterns, crowd density, and environmental conditions. By doing so, we ensure that surveillance systems remain effective under a wide range of scenarios, thereby enhancing security measures in public spaces. Furthermore, real-time adaptability positions them at the forefront of surveillance technology research, offering potential pathways for future innovations in the field. Moreover, AI is able to offer a sophisticated approach to surveillance that promises not only improved efficiency and adaptability but also a model for future research and development in the realm of security technologies. As the world continues to navigate the complexities of public safety and security, the findings of this research provide valuable insights and tools for enhancing surveillance measures in an ever-changing global landscape.

Based on the above observations and solid motivations, the main contributions of this paper are summarized as follows:

- To the best of our knowledge, this is the first approach to apply deep learning-enabled surveillance in smart mobility environments and dynamic cyber-physical spaces with mobile elements. This study introduces a novel approach that leverages the capabilities of deep learning to enhance surveillance systems’ responsiveness to dynamic environments. In this study, we construct deep learning-enabled surveillance barriers considering obstacle avoidance in the given area.
- The proposed system seeks to bridge the aforementioned gaps by proposing an innovative extension of the K-barrier model through the integration of deep learning technologies, aiming to enhance the adaptability and effectiveness of surveillance systems when faced with unpredictable human behavior.
- We define the problem, whose main objective is to maximize the number of deep learning-assisted surveillance barriers for smart mobility. Then, two different algorithms are devised to resolve the problem. Furthermore, their performance is analyzed through extensive simulations under various scenarios, and a detailed discussion is presented.

This paper is organized as follows. Section 2 introduces the proposed deep learning-assisted surveillance reinforcement system for dynamic environments and formally defines the main research problem. Then, to solve the defined problem, two different algorithms are presented in Section 3. Furthermore, the performance of the developed schemes is demonstrated through a detailed discussion in Section 4, based on the outcomes obtained through extensive experiments. Finally, this paper concludes in Section 6.

## 2. Proposed System

The proposed system not only addresses the immediate challenges posed by dynamic environments but also sets the stage for the development of future surveillance technologies. The implications of this study extend beyond the technical advancements in surveillance systems, touching on the broader themes of privacy, ethics, and the societal impact of increasingly autonomous security measures. Also, this study introduces a novel approach that leverages the capabilities of deep learning to enhance surveillance systems’ responsiveness to dynamic environments. Deep learning, with its unparalleled ability to process and learn from large datasets, offers a promising solution to the limitations of traditional surveillance models.

### 2.1. System Overview

The system is uniquely designed to adapt to dynamic environments by incorporating predictive models that estimate population density based on input weather and temperature conditions. It follows that the proposed system focuses on a surveillance system tailored for public spaces, referred to as *stations*, which include but are not limited to airports, train stations, shopping centers, and smart homes. After the sensors or devices are initially randomly distributed in the given area, the proposed framework allows for an intelligent and dynamic adaptation of surveillance strategies in real time, catering to the unique challenges presented by human mobility and environmental conditions. The critical components and functionalities of this system are outlined below.

#### 2.1.1. Station

We define the operational environment for the surveillance system, encompassing various public spaces with significant human activity and mobility. These environments require sophisticated surveillance mechanisms due to their dynamic nature.

#### 2.1.2. Weather

This surveillance system incorporates a nuanced approach by using weather and temperature conditions input by the user to estimate the population density within the station. Specifically, the system’s predictive model makes use of a weather condition scale ranging from 1 to 5, with lower numbers indicating poorer weather conditions. This scale is pivotal for understanding how variations in weather and temperature significantly impact the number of people visiting the station. For instance, bad weather conditions (represented by lower values on the scale) are expected to reduce station footfall, whereas better conditions (indicated by higher values) are expected to lead to an increase in population density. By integrating this relationship into the predictive model, the system can dynamically adjust surveillance strategies to align with anticipated changes in population density due to varying weather conditions. Such adaptability is essential for optimizing surveillance coverage and ensuring that the system remains effective across diverse environmental scenarios.

#### 2.1.3. Pedestrians

From the surveillance system’s perspective, people within the station are viewed as obstacles that can affect surveillance effectiveness. The system employs predictive analytics to understand and anticipate the dynamic movement patterns of people, adjusting sensor placements accordingly.

### 2.2. Deep Learning Model

This study employs a Recurrent Neural Network (RNN)-based model that leverages Long Short-Term Memory (LSTM) units to enhance efficiency and adaptability in dynamic environments. The choice of RNNs, and more so LSTMs, is due to their proficiency in analyzing and predicting complex temporal patterns in data, which are crucial for interpreting the dynamics of population movement, environmental changes, and other relevant variables in real time.

#### 2.2.1. Model Architecture

The architecture of our LSTM-based model is designed to capture and analyze the temporal dependencies and patterns inherent in surveillance data. It follows that this architecture allows for a deeper understanding of temporal sequences, facilitating more accurate and timely predictions in surveillance applications. Unlike traditional neural networks, LSTMs have the unique ability to remember information for long periods, which is crucial for predicting future crowd movements and detecting anomalies in behavior over time. The model consists of the following layers:

- An LSTM layer with 20 units and a tanh activation function to capture the temporal dependencies within the data.
- A dense layer with 15 units and a tanh activation function for further processing of the LSTM outputs.
- A dense output layer to predict the average number of barriers.

#### 2.2.2. Activation Function

The tanh activation function, which is used in the LSTM’s gates, helps regulate the flow of information by scaling the output values between −1 and 1. This is crucial for maintaining the stability of the gradients during backpropagation and allowing the model to learn long-term dependencies efficiently. The *tanh* activation function is defined as

(1) $$\tanh\left(x\right)=\frac{e^{x}-e^{-x}}{e^{x}+e^{-x}}$$

#### 2.2.3. Cost Function

The model employs the Mean Squared Error (MSE) as its cost function to quantify the difference between the predicted average number of barriers and the actual data:

(2) $$\mathrm{MSE}=\frac{1}{n}\sum_{i=1}^{n}{\left(y_{i}-{\hat{y}}_{i}\right)}^{2}$$

#### 2.2.4. Input

The model takes the following inputs, which are pivotal for tailoring the surveillance response to environmental and operational conditions:

- Weather Conditions: These impact crowd behavior and surveillance requirements; e.g., inclement weather may reduce outdoor activity.
- Area Size: This affects the distribution and density of crowds—the density of people within stations for the purpose of ticket counts and check-in—which are important for deploying resources efficiently.
- Sensing Radius: This determines the coverage area of sensors, which is crucial for optimal surveillance.
- Lifetime: This indicates maintenance needs, ensuring uninterrupted operation.
- Number of Sensors: The number of sensors is an important variable that determines how well they can cover the monitored area and the amount and quality of data the system can collect.
- Different Time-Series Types: Regular, pre-holiday, and holiday seasons can be considered in transportation stations managed by municipal authorities, railways, and airports.

#### 2.2.5. Output

The main output of this model is the prediction of the average number of barriers over a specific period. This is important for helping the surveillance system determine the optimal number of sensors required for each time unit and to ensure the coverage and surveillance efficiency required within the surveillance area.

#### 2.2.6. Training and Validation

Training the LSTM model involves using historical data to learn the sequential dependencies of the observed variables, including crowd density, movement patterns, and other relevant temporal factors. The model is trained through a supervised learning process, where it learns to predict the next sequence in the data, such as future crowd density, based on past observations. A validation dataset is utilized to tune the model’s parameters and avoid overfitting, ensuring its robustness and reliability in real-world scenarios. During the validation phase, the model’s predictive performance is rigorously evaluated against unseen data, ensuring its ability to generalize across different times and conditions. This step is critical for assessing the model’s practical applicability and for making necessary adjustments to improve its accuracy and effectiveness.

#### 2.2.7. Continuous Learning and Adaptation

A key feature of our model is its capacity for continuous learning, allowing it to adapt to new patterns and changes in the environment. This ongoing learning process is facilitated by periodically updating the model with new data, ensuring that its predictions remain accurate over time. By adapting to new data, the model helps maintain the surveillance system’s effectiveness, even as the dynamics of the monitored spaces evolve.

#### 2.2.8. Problem Definition

**Definition** **1** (Deep learning-assisted surveillance barrier maximization problem)**.** *Given the set of station sensors S and the set of obstacles T in the station area A, the deep learning-assisted surveillance barrier maximization problem, referred to as* MaxDLSurv, *aims to maximize the number of deep learning-assisted surveillance barriers β such that obstacles, pedestrians, and weather conditions are considered.*

## 3. The Proposed Schemes

This section presents two schemes devised to resolve the *MaxDLSurv* problem.

### 3.1. Total-Space Coverage: TSC

**Figure 4:**
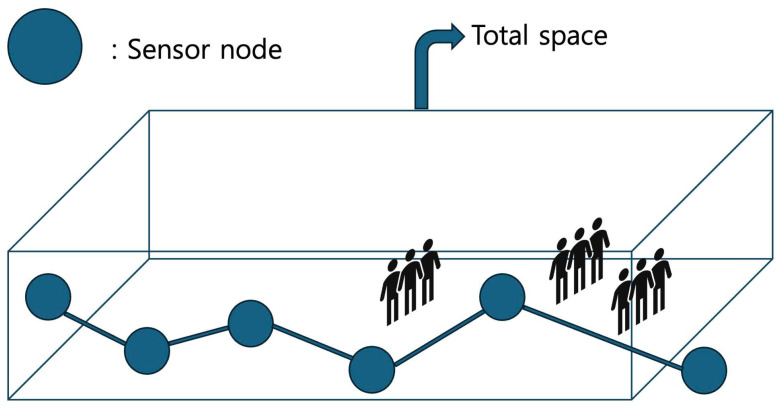
The total-space-coverage strategy of the *TSC* algorithm.

The *total-space-coverage* algorithm, called *TSC*, aims to cover the entire area while considering humans as obstacles. This strategy involves deploying sensors across the entire area to ensure comprehensive surveillance coverage. The main goal of the *total-space-coverage* algorithm is to maximize the number of deep learning-assisted surveillance barriers β such that the obstacles in the area are considered. Figure 4 shows the strategy of the *total-space-coverage* scheme. The pseudo-code for *TSC* is presented in Algorithm 1, along with detailed descriptions.
**Algorithm 1** ***Total-Space Coverage*** Input: S,T,A, Output: β1:initialize an empty list for station sensor placements;2:**for** each point in station area *A* **do**3:    **if** point is not in obstacles *T* **then**4:         please sensor at point;5:         add point to sensor placements;6:use LSTM model to decide sensor placements;7:estimate the total number of decided placements as β;8:return β;

### 3.2. Subspace Coverage: SSC

**Figure 5:**
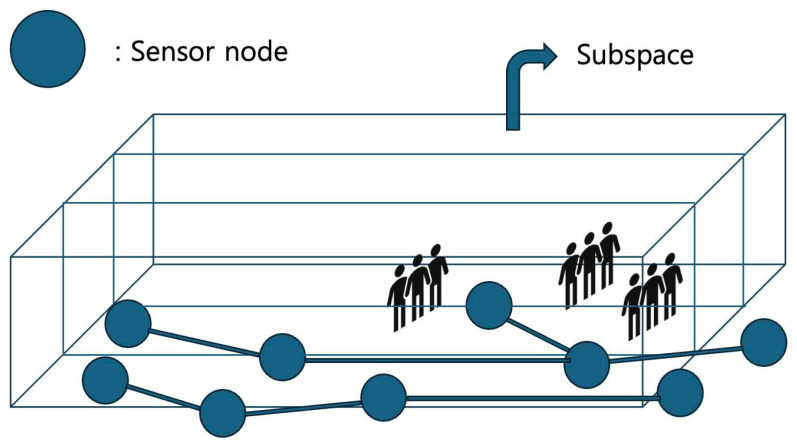
The execution of the *SSC* algorithm.

The basic idea of the *subspace-coverage* algorithm, referred to as *SSC*, is to divide the area *A* into smaller segments for more targeted surveillance coverage. The total number of barriers is the sum of barriers β needed for each segmented space. The main objective of the *subspace-coverage* algorithm is to maximize the number of deep learning-assisted surveillance barriers β. Figure 5 shows the execution of the *SSC* algorithm. Also, the pseudo-code for *SSC* is presented in Algorithm 2, along with detailed specifications.
**Algorithm 2** ***Subspace Coverage*** Input: S,T,A, Output: β1:determine the number of segments required;2:divide station area *A* into segments based on requirements;3:initialize total barriers to 0;4:**for** each segment in *A* **do**5:     , estimate potential barrier formations for segment with *T*;6:     use LSTM model to predict complete barrier formations for segment;7:     add the number of barriers for segment into β;8:return β;

## 4. Experimental Analysis

When exploring the deployment of an LSTM-based deep learning model to enhance surveillance systems through dynamic barrier placement, some important insights were obtained. The model’s performance was evaluated through a series of metrics, underscoring the potential of integrating advanced machine learning techniques into real-world applications.

### 4.1. Training and Validation Outcomes

**Figure 6:**
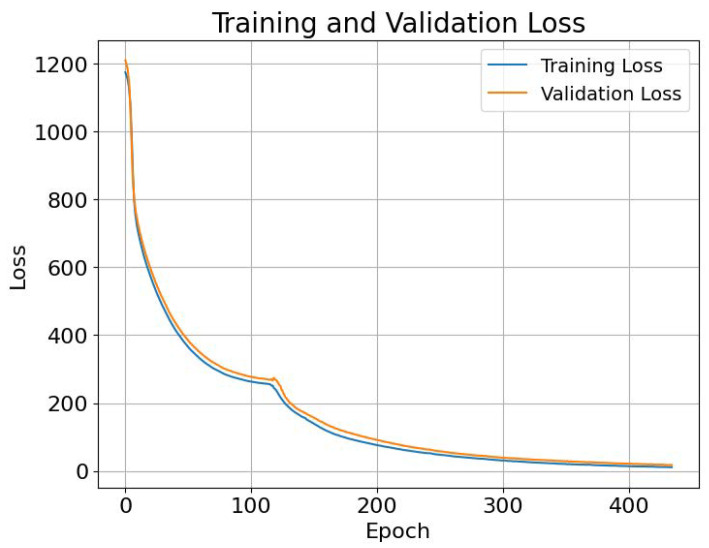
Comparison between the training loss and validation loss.

The training phase concluded with a loss of 11.0841, reflecting the model’s efficiency in learning from the training dataset, which included various features such as the area size, weather conditions, sensing radius, number of nodes, and device lifetime. In parallel, the validation loss was recorded at 17.7. This discrepancy between the training and validation loss suggests potential issues with generalization, which is a focal point for further investigation and optimization. Figure 6 depicts the comparison between the training loss and validation loss.

### 4.2. Testing Phase Performance

Upon evaluating the model against the test dataset, a test loss of 17.3 was achieved. This result aligned closely with the validation loss, indicating consistent model performance when exposed to unseen data. The test phase outcomes serve as a testament to the model’s potential utility in predicting optimal barrier placements in surveillance systems under varying conditions.

### 4.3. Computational Efficiency

A noteworthy aspect of the model’s implementation was its computational efficiency, with the total training time amounting to approximately 36.52 s. This rapid training process not only demonstrates the model’s suitability for real-time applications but also highlights its potential for deployment in scenarios where timely decision-making is crucial.

### 4.4. Discussion

Our experiments were conducted in a Google Colab environment utilizing Tesla V100 GPUs. The dataset used was adapted from the experimental results of existing algorithms. The evaluation was divided into three experimental groups, each analyzing the performance of the *TSC* and *SSC* algorithms under different spatial sizes, lifetimes, and weather conditions.

**Figure 7:**
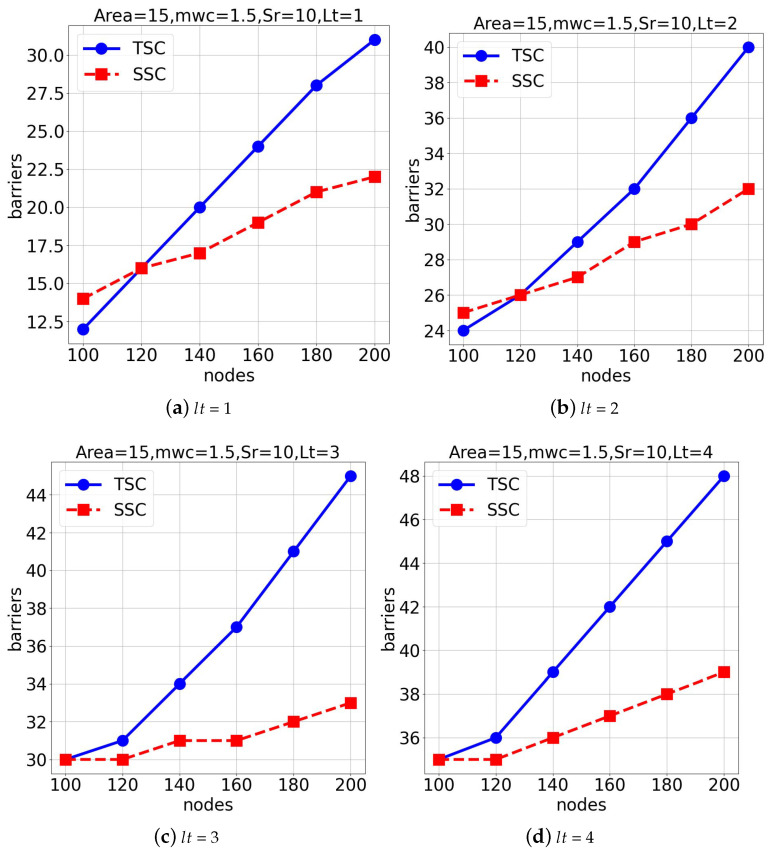
Performance comparison under different lifetimes (lt) in a 1500×1500×1500 space.

In the first experimental group, we evaluated both algorithms in a space of 1500×1500×1500 under average weather conditions (mwc = 1.5), as seen in Figure 7. The aim of this experiment was to observe the number of deep learning-assisted surveillance barriers generated as the node or sensor count increased. Figure 7a,b show the results for lifetimes (lt) of 1 and 2. Also, Figure 7c,d present the outcomes for lifetimes (lt) of 3 and 4, respectively. The *TSC* algorithm generally outperformed the *SSC* algorithm under these settings.

**Figure 8:**
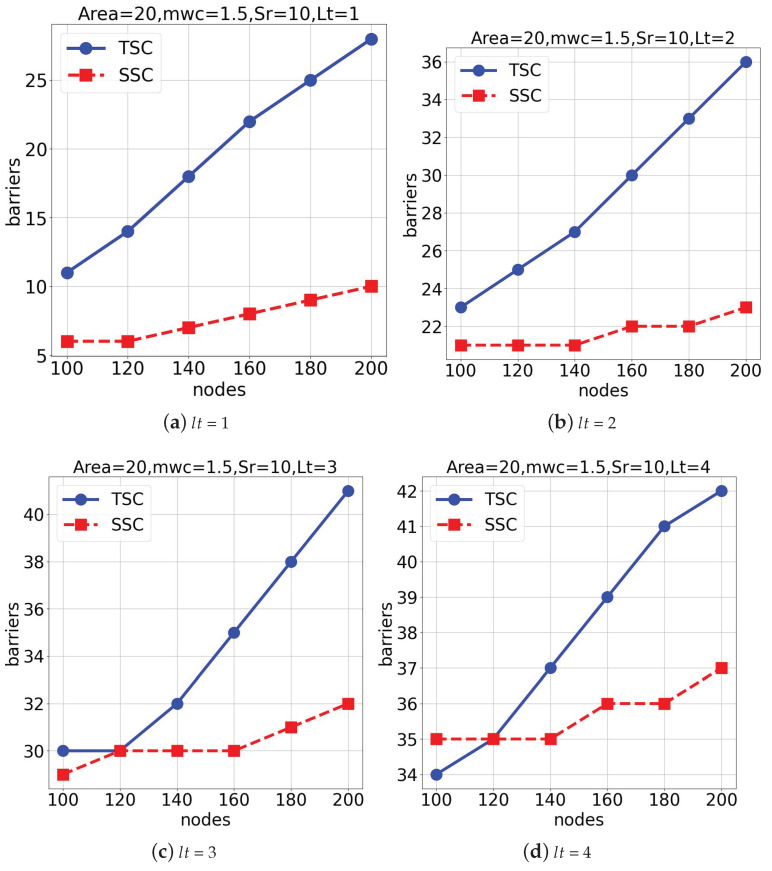
Performance comparison under different lifetimes (lt) in a 2000×2000×2000 space.

In the second set of experiments, both schemes were executed in a space of 2000×2000×2000 under average weather conditions (mwc = 1.5), as shown in Figure 8. Similar to the first set of simulations, the number of deep learning-assisted surveillance barriers increased as the total number of nodes increased. Also, Figure 8a,b represent the outcomes when lifetimes (lt) of 1 and 2 were considered. Figure 8c,d show the results when lifetimes (lt) of 3 and 4 were considered. From Figure 8, we can conclude that the *TSC* algorithm outperformed the *SSC* algorithm.

**Figure 9:**
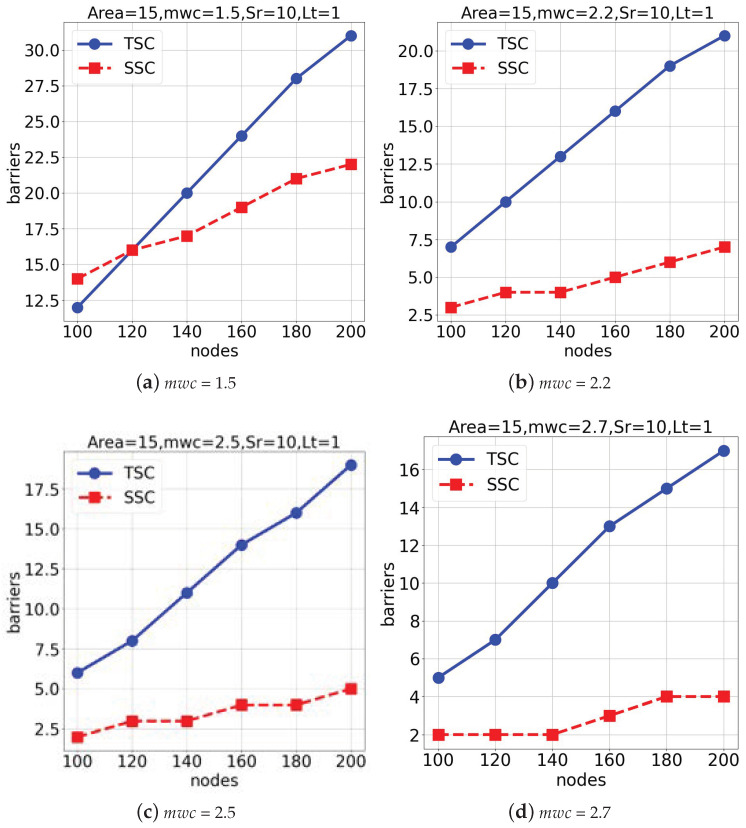
Performance comparison under various weather conditions (mwc) in a 1500×1500×1500 space.

The third group of experiments was conducted in a 1500×1500×1500 space with a lifetime of 1 to evaluate the model’s performance under varying weather conditions (mwc), as shown in Figure 9. Figure 9a,b represent the results obtained when mwc = 1.5 and 2.2. Also, Figure 9c,d show the outcomes when mwc = 2.5 and 2.7. The *TSC* algorithm demonstrated superior performance over the *SSC* algorithm across all tested weather conditions. Overall, the *TSC* algorithm consistently outperformed the *SSC* algorithm across various environments and conditions. The adaptability and efficiency of the *TSC* algorithm, especially in response to changes in the space size and weather conditions, demonstrate its robustness and effectiveness.

## 5. Future Work

### 5.1. Statistical Metrics and Comparisons

In future work, we will extend the experiments by considering additional statistical metrics or error visualization and provide comparisons of random versus uniform placement of sensors.

### 5.2. Cross-Validation in a Real Testbed

It is necessary to expand the simulations with cross-validation and to provide public availability of the dataset collected from real testbed experiments with practical scenarios.

## 6. Concluding Remarks

This study focused on the development of a deep learning-enhanced surveillance model for big data-based smart mobility environments and dynamic cyber-physical spaces with mobile elements. We designed the surveillance system to efficiently divide the three-dimensional space into planes to extend the theory of the maximization of two-dimensional barriers to three dimensions. To divide the space into planes, a dividing criterion was required; the dividing criterion was proposed as a solution, and the number of deep learning-assisted surveillance barriers was estimated as a return value. With a formal definition of *MaxDLSurv*, two different schemes were proposed. The experiments conducted in this study conclusively demonstrated that the *TSC* algorithm consistently outperformed the *SSC* algorithm across various environments and conditions.

## Data Availability

The data presented in this study are available on request from the corresponding author.

## Abbreviations

The following abbreviations are used in this manuscript:

IoT	Internet of Things
IIoT	Industrial Internet of Things
